# A comparative evaluation of dexmedetomidine and midazolam in pediatric sedation: A meta‐analysis of randomized controlled trials with trial sequential analysis

**DOI:** 10.1111/cns.13377

**Published:** 2020-04-29

**Authors:** Bingchen Lang, Lingli Zhang, Wensheng Zhang, Yunzhu Lin, Yuzhi Fu, Shouming Chen

**Affiliations:** ^1^ Department of Pharmacy West China Second University Hospital Sichuan University Chengdu China; ^2^ Key Laboratory of Birth Defects and Related Diseases of Women and Children Ministry of Education Sichuan University Chengdu China; ^3^ Evidence‐Based Pharmacy Center West China Second University Hospital Sichuan University Chengdu China; ^4^ Laboratory of Anesthesia and Critical Care Medicine Department of Anesthesiology Translational Neuroscience Center West China Hospital Sichuan University Chengdu China; ^5^ Department of Anesthesiology West China Second University Hospital Sichuan University Chengdu China

**Keywords:** dexmedetomidine, meta‐analysis, midazolam, pediatrics, sedation

## Abstract

**Background:**

The present study with trial sequential analysis (TSA) was conducted to evaluate comprehensively the efficacy and safety of dexmedetomidine and midazolam in pediatric sedation, and to investigate whether the outcomes achieved the required information size to draw the conclusions.

**Methods:**

PubMed, Embase, and Cochrane Library were searched from inception to October 2019. All randomized controlled trials used dexmedetomidine and midazolam in pediatric sedation were enrolled. Sedative efficacy, postoperative analgesic effect, and incidence of emergence agitation were considered as the co‐primary outcomes. Grading of Recommendations Assessment, Development, and Evaluation (GRADE) system was applied to rate the quality of evidences.

**Results:**

We acquired data from 34 studies involving 2281 pediatric patients. The results indicated that administration of dexmedetomidine was associated with less incidence of emergence agitation (RR = 0.78, with 95% CI [0.65, 0.92]) and more satisfactory sedation at parental separation (RR = 0.31, with 95% CI [0.24, 0.41]) compared to midazolam, and the current sample sizes were sufficient with unnecessary further trials. Two groups did not differ significantly in sedation level at mask induction (RR = 0.86, with 95% CI [0.74, 1.00]). And using of dexmedetomidine was associated with less incidence of postoperative analgesic rescue (RR = 0.57, with 95% CI [0.35, 0.93]), but the number of patients was too few to achieve the required information size and to draw reliable conclusions. Premedication of dexmedetomidine was associated with significant less value of SBP, heart rate, increased incidence of bradycardia, and a lower rate of shivering. And there were no differences about onset of sedation and recovery time between two groups.

**Conclusions:**

Given that more satisfactory sedation at separation from parents and less incidence of emergence agitation, dexmedetomidine is preferred for pediatric sedation. However, compared with midazolam, the superiority of dexmedetomidine in providing adequate sedation at mask induction and postoperative analgesic effects has not yet been defined.

## 1 INTRODUCTION

Anxiety and distress developed in pediatric patients during perioperative period bring the challenges for anesthesiologist and pediatric clinicians. Uncooperative physically resistance from children results in increased difficulties in separation from parents, mask application, and induction of anesthesia.^1^, ^2^ It is estimated that up to 60%‐70% of children suffered anxiety, anguish, and fear throughout the perioperative period or diagnostic procedures.^3^ Sedative premedications can help to reduce the anxiety, minimize the emotional discomforts, ease the parental separation, and smooth the induction of anesthesia.^4^ Many premedicants via different routes have been tried in clinical practice.

Compared with other benzodiazepine, midazolam has rapid onset and high metabolic clearance, and its sedative efficacy in pediatric premedication has been demonstrated widely. However, untoward effects including negative postoperative behavioral changes, cognitive impairment, respiratory depression, and insufficient prevention of postoperative emergence agitation have been reported in children premedicated with midazolam,^5^, ^6^ which makes it a less‐than‐ideal option in pediatric sedation.

As one highly selective α2‐adrenoceptor agonist (selectivity ratio for α2‐adrenoceptor:α1‐adrenoceptor is 1600:1) with sedative and analgesic characteristics, dexmedetomidine provides cooperative and arousable sedation without clouded consciousness and respiratory depression. Owing to these beneficial effects, it has been demonstrated to be a useful pre‐anesthesia medication in children.^7^

In an effort to evaluate the influences of the two premedications on pediatric perioperative sedation, Pasin et al^8^ and Sun et al^9^ conducted the relevant meta‐analyses with total of 13 randomized trials (1033 patients) and 11 randomized trials (829 patients) which showed a satisfactory sedation profiles of dexmedetomidine premedication. The included items and the sample size of two studies were approximate, and authors also described that available data were still lacking. More evidences with large sample size were required to draw the reliable conclusions.

Therefore, on the basis of combining the latest evidences, the present updated meta‐analysis of RCTs was conducted to evaluate comprehensively the effects of two premedicants in pediatric sedation at separation from parents and mask induction, hemodynamic status, and various adverse effects. And the trial sequential analysis (TSA) was also performed to determine whether the findings achieved the required information size to draw the conclusions.

## 2 MATERIALS AND METHODS

The present meta‐analysis was performed in accordance with the recommendations in the Preferred Reporting Items for Systematic Reviews and Meta‐Analyses (PRISMA) statement^10^ and the guidelines described in the *Cochrane Handbook*.

### 2.1 Search strategy

Two independent reviewers (BL and YF) performed the literature search. The databases including PubMed, Embase, and Cochrane Library were searched systematically. The strategies used for searching were including infant, child, adolescent, dexmedetomidine, midazolam, and randomized controlled trial (Appendix S1). Only human studies were involved, and there were no restrictions of language. The final literature search was performed on October 7, 2019.

### 2.2 Eligibility criteria

The studies meeting the following criteria were selected for further analysis:

#### 2.2.1 Participants

The patients were the children (<18 years old) who experienced different surgical and diagnostic procedures.

#### 2.2.2 Intervention and comparison

Using dexmedetomidine vs midazolam as the premedication (regardless of the route and dose of administration).

#### 2.2.3 Outcome measures

Given that satisfactory separation from parents and satisfactory induction or facemask compliance with limited postoperative pain and agitation were considered as the ideal characteristics of pediatric sedatives,^9^ the co‐primary outcomes were as follows: (a) number of patients with satisfactory separation from parents, (b) number of patients with satisfactory induction or mask acceptance, (c) number of patients requiring postoperative analgesics rescue, and (d) incidence of emergency agitation. The general hemodynamic parameters, onset of sedation, and recovery time between two groups were considered as the secondary outcomes. The incidence of adverse events, including shivering, bradycardia, nausea, and vomiting, were also analyzed.

#### 2.2.4 Study design

Randomized controlled trials with no language limitations.

### 2.3 Literature screening, data extraction, and assessment of the risk of bias

Two reviewers (BL and YF) conducted the literature searching and data extraction independently, and then they cross‐checked with each other. After removing the duplicates from different databases, those obviously irrelevant records were excluded by titles and abstracts reviewing. The full texts of the remaining studies were obtained and perused. And then, the relevant articles were identified. To collect the general characteristics of enrolled studies, a table was designed and filled by us (Table 1). In accordance with Cochrane Collaboration tool for assessing risk of bias in randomized trials,^11^ two reviewers (BL and YF) independently evaluated the methodological quality which includes the following aspects: random sequence generation, allocation concealment, blinding of participants and personnel, blinding of outcome assessment, incomplete outcome data, and selective reporting. Any disagreements were resolved by consensus through discussion among all authors.

**Table 1:** The general characteristics of the enrolled studies

Study (Reference)	Year	Type of surgery/procedure	Patient age (range) & ASA status	Patients enrolled (Gender: F/M, n)	DEX dose, route of administration	MDZ dose, route of administration	Scale used for sedation measurement	Outcomes
Tobias et al^14^	2004	Mechanical ventilation	Not mentioned	30 (10/20) 1. DEX 0.25 patients: 10 2. DEX 0.5 patients: 10 3. MDZ patients: 10	1. DEX 0.25: 0.25 μg/kg/h, Infusion; 2. DEX 0.5: 0.5 μg/kg/h, Infusion	0.1 mg/kg/h, Infusion	6‐points scale	V, VI, VIII,
Koroglu et al^15^	2005	Magnetic resonance imaging procedures	1‐7 y (ASA I‐II)	80 (29/51) 1. DEX patients: 40 2. MDZ patients: 40	Loading dose 1 μg/kg followed by 0.5 μg/kg/h, Infusion	Loading dose 0.2 mg/kg followed by 6 μg/kg/h, Infusion	6‐points scale	VII‐X
Schmidt et al^16^	2007	Elective ambulatory surgical procedures	7‐12 y (ASA I‐II)	42 (18/24) 1. DEX patients: 20 2. MDZ patients: 22	1 μg/kg, Oral transmucosal	0.5 mg/kg, Oral	4‐point scale	III, VII, VIII, X,
Yuen et al^17^	2008	Elective minor surgery	2‐12 y (ASA I‐II)	96 (7/89) 1. DEX 0.5 patients: 32 2. DEX 1.0 patients: 32 3. MDZ patients: 32	1. DEX 0.5 patients: 0.5 μg/kg, Intranasal; 2. DEX 1.0 patients: 1 μg/kg, Intranasal	0.5 mg/kg, Oral	6‐points scale	I, II
Talon et al^18^	2009	Elective reconstructive surgery	1‐18 y (Not mentioned)	100 (47/53) 1. DEX patients: 50 2. MDZ patients: 50	2 μg/kg, Intranasal	0.5 mg/kg, Oral	6‐points scale	I‐III
Aksu et al^19^	2011	Electroencephalogram procedures	6 mo‐6 y (ASA I)	60 (18/42) 1. DEX patients: 30 2. MDZ patients: 30	0.5 μg/kg, IV	0.1 mg/kg, IV	6‐points scale	IV, X
Ghali et al^20^	2011	Elective outpatient adenotonsillectomy surgery	4‐12 y (ASA I)	120 (58/62) 1. DEX patients: 60 2. MDZ patients: 60	1 μg/kg, Intranasal	0.5 mg/kg, Oral	6‐points scale	I, III, V, VIII, X
Mountain et al^21^	2011	Dental restoration and possible tooth extraction	1‐6 y (ASA I)	41 (20/21) 1. DEX patients: 22 2. MDZ patients: 19	4 μg/kg, Oral	0.5 mg/kg, Oral	4‐point scale	I, II, IV
Özcengiz et al^22^	2011	Esophageal dilatation procedures	3‐9 y (ASA I‐II)	50 (26/24) 1. DEX patients: 25 2. MDZ patients: 25	2.5 μg/kg, Oral	0.5 mg/kg, Oral	Not mentioned	IV
Akin et al^23^	2012	Elective adenotonsillectomy	2‐9 y (ASA I)	90 (37/53) 1. DEX patients: 45 2. MDZ patients: 45	1 μg/kg, Intranasal	0.2 mg/kg, Intranasal	6‐points scale	I‐IV, XII
Aydogan et al^24^	2013	Scoliosis surgery	12‐18 y (ASA I‐II)	32 (15/17) 1. DEX patients: 16 2. MDZ patients: 16	0.4 μg/kg/h, Infusion	0.1 mg/kg/h, Infusion	Richmond Agitation Sedation Scale	IV, XI
Bhadla et al^25^	2013	Ophthalmic day‑care surgery	5‐12 y (ASA I‐II)	60 (21/39) 1. DEX patients: 30 2. MDZ patients: 30	0.4 μg/kg, IV	0.05 mg/kg, IV	5‐points scale	I, II, IV, V, VIII, XIII
Sheta et al^26^	2013	Complete dental rehabilitation	3‐6 y (ASA I‐II)	72 (41/31) 1. DEX patients: 36 2. MDZ patients: 36	1 μg/kg, Intranasal	0.2 mg/kg, Intranasal	4‐point scale	I‐IV, IX, X, XII, XIII
Arora et al^27^	2014	Elective urogenital surgical procedures	1‐4 y (ASA I‐II)	56 (4/52) 1. DEX patients: 27 2. MDZ patients: 29	4 μg/kg, Oral	0.5 mg/kg, Oral	4‐point scale	I, II
Pant et al^28^	2014	Inguinal hernia repair, orchidopexy, or circumcision	1‐12 y (ASA I‐II)	100 (12/88) 1. DEX patients: 50 2. MDZ patients: 50	1.5 μg/kg, Sublingually	0.25 mg/kg, Sublingually	6‐point scale	I, II
Savla et al^29^	2014	Short elective surgical procedure	1‐6 y (ASA I‐II)	34 (2/32) 1. DEX patients: 19 2. MDZ patients: 15	2 μg/kg, Intranasal	0.5 mg/kg, Intranasal	6‐point scale	II
Linares Segovia et al^30^	2014	Elective surgery	2‐12 y (ASA I)	108 (52/56) 1. DEX patients: 52 2. MDZ patients: 56	1 μg/kg, Intranasal	0.5 mg/kg, Oral	N/A	II, IV
Surendar et al^31^	2014	Dental treatment	4‐14 y (ASA I)	63 (N/A) 1. DEX 1.0 patients: 21 2. DEX 1.5 patients: 21 3. MDZ patients: 21	1. DEX 1.0 patients: 1 μg/kg, Intranasal; 2. DEX 1.5 patients: 1.5 μg/kg, Intranasal	0.5 mg/kg, Intranasal	5‐points scale	V, VI, VIII‐X
Hojjat et al^32^	2015	Computed tomography scan procedures	2‐12 y (Not mentioned)	100 (44/56) 1. DEX patients: 50 2. MDZ patients: 50	2 μg/kg, IV	0.05 mg/kg, IV	6‐point scale	IV
Faritus et al^33^	2015	Surgery for congenital heart disease	2‐12 y (Not mentioned)	60 (28/32) 1. DEX patients: 30 2. MDZ patients: 30	2 μg/kg, Oral	0.5 mg/kg, Oral	6‐point scale	II, V, VI, VIII
Singla et al^34^	2015	Elective surgery	3‐10 y (ASA I)	60 (29/31) 1. DEX patients: 30 2. MDZ patients: 30	1 μg/kg, Intranasal	0.5 mg/kg, Intranasal	6‐point scale	I, II, V, VIII
Abdelaziz et al^35^	2016	Elective strabismus surgery	1‐7 y (ASA I‐II)	66 (32/34) 1. DEX patients: 33 2. MDZ patients: 33	1 μg/kg, Intranasal	0.1 mg/kg, Intranasal	N/A	III, IV, XII
Ghai et al^36^	2016	Computed tomography scan procedures	1‐6 y (ASA I‐II)	59 (36/23) 1. DEX patients: 30 2. MDZ patients: 29	2.5 μg/kg, Intranasal	0.5 mg/kg, Oral	6‐point scale	I
Jambure et al^37^	2016	Cardiac catheterization for diagnostic/therapeutic procedures	2‐10 y (ASA I‐II)	61 (15/46) 1. DEX patients: 31 2. MDZ patients: 30	2 μg/kg, Intranasal	0.5 mg/kg, Oral	6‐point scale	IX, XI
Jannu et al^38^	2016	Elective, minor, lower abdominal surgeries	1‐7 y (ASA I‐II)	60 (32/28) 1. DEX patients: 30 2. MDZ patients: 30	4 μg/kg, Oral	0.75 mg/kg, Oral	4‐point scale	IV, IX,
Gupta et al^39^	2017	Elective brain magnetic resonance imaging	1‐8 y (ASA I‐II)	60 (26/34) 1. DEX patients: 30 2. MDZ patients: 30	1 μg/kg, Intranasal	0.2 mg/kg, Intranasal	6‐point scale	I, II
Prabhu et al^40^	2017	Elective surgery	1‐10 y (ASA I‐II)	90 (35/55) 1. DEX patients: 45 2. MDZ patients: 45	4 μg/kg, Oral	0.5 mg/kg, Oral	N/A	II‐IV, X
Kumar et al^41^	2017	Abdominal surgery	2‐12 y (ASA I‐II)	60 (26/34) 1. DEX patients: 30 2. MDZ patients: 30	1 μg/kg, Intranasal	0.5 mg/kg, Oral	6‐point scale	I, II
Kumari et al^42^	2017	Ophthalmic surgery	4‐12 y (ASA I)	60 (25/35) 1. DEX patients: 30 2. MDZ patients: 30	4 μg/kg, Oral	0.5 mg/kg, Oral	3‐point scale	I, II
Li et al^43^	2017	Selective primary repair for tetralogy of Fallot	5‐28 mo (Not mentioned)	38 (N/A) 1. DEX patients: 20 2. MDZ patients: 18	5 μg/kg, Oral	0.5 mg/kg, Oral	5‐points scale	I, II, IX
Surana et al^44^	2017	Cleft palate surgery	6 mo‐12 y (ASA I)	60 (30/30) 1. DEX patients: 30 2. MDZ patients: 30	Loading dose 1 μg/kg followed by 0.5 μg/kg/h, Infusion	0.05 mg/kg, IV	N/A	III, XI
Abdel‐Ghaffar et al^45^	2018	Bone marrow aspiration and biopsy	3‐7 y (ASA I‐II)	60 (31/29) 1. DEX patients: 30 2. MDZ patients: 30	2 μg/kg, Inhalation	0.2 mg/kg, Inhalation	5‐points scale	I, II, IV, XII
Sajid et al^46^	2019	Elective herniotomy	1‐6 y (ASA I)	80 (32/48) 1. DEX patients: 40 2. MDZ patients: 40	4 μg/kg, Oral	0.5 mg/kg, Oral	5‐points scale	I, II, IV, XI,
Sathyamoorthy et al^47^	2019	Dental procedures	5‐18 y (Not mentioned)	73 (23/50) 1. DEX patients: 36 2. MDZ patients: 37	0.2 μg/kg, Intranasal	0.5 mg/kg, Oral	5‐points scale	I, II

Note

I—Number of patients with satisfactory separation from parents; II—Number of patients with satisfactory induction or mask acceptance; III—Incidence of postoperative pain needed analgesics rescue; IV—Incidence of emergence agitation; V—Hemodynamic status (SBP); VI—Hemodynamic status (DBP); VII—Hemodynamic status (MAP); VIII—Hemodynamic status (HR); IX—Onset of sedation; X—Recovery time; XI—Incidence of adverse events (Bradycardia); XII—Incidence of adverse events (Nauseas and vomiting); XIII—Incidence of adverse events (Shivering).

Abbreviations: ASA, American Society of Anesthesiologist physical status; DBP, Diastolic blood pressure; DEX, Dexmedetomidine; HR, Heart rate; MAP, Mean arterial pressure; MDZ, Midazolam; SBP, systolic blood pressure.

### 2.4 Grading the quality of evidence

The Grading of Recommendations Assessment, Development, and Evaluation (GRADE) methodology was used to assess the quality of evidence and strength of recommendations. The quality of all primary and secondary outcomes was independently assessed by two reviewers (BL and YF). On the basis of risk of bias, inconsistency, indirectness, imprecision, and publication bias, the quality was classified as high, moderate, low, or very low.^12^ And GRADE profiler (version 3.6) software was used.

### 2.5 Statistical analysis

Statistical analyses were done with Review Manager 5.0 software (The Cochrane Collaboration). The risk ratio (RR) with 95% confidence interval (CI) and the Mantel‐Haenszel method (fixed or random models) were used to analyze dichotomous data. For continuous data, standardized mean difference (SMD) was chosen for the estimation. The *I*‐squared (*I* ^2^) test was chosen to weigh the impact of heterogeneity on the results. If significant heterogeneity (present at *I* ^2^ > 50%) emerged, the sensitivity analysis was performed by omitting each study individually, and the random effects model was chosen; otherwise, the fixed‐effects model was applied. Publication bias was evaluated by using Begg's test when approximate ten studies or more were included in meta‐analysis. A *P* value <.05 was considered statistically significant.

### 2.6 Trial sequential analysis

The sparse data and the repetitive significance testing with new studies updating may result in type‐1 errors (false‐positive outcomes) and type‐2 errors (false‐negative outcomes) of meta‐analyses. Trial sequential analysis (TSA), which controlled the *P* value and widen the confidence intervals, can adjust the statistical threshold to decrease or eliminate the risk from type‐1 and type‐2 errors, and can estimate the required information size and trial sequential monitoring boundaries. The cumulative Z curve entering the futility area or crossing the trial sequential monitoring boundary may indicate that the present evidences of intervention effects are at a sufficient level, and further trials will be unnecessary. On the contrary, evidences are insufficient to arrive at the conclusion if Z curve does not cross any boundaries or reach the required information size.^13^ The type‐I error (*α*) and power were set as 0.05 and 0.80, respectively. For the same outcome, all relevant trials would be involved in analysis, and the results would not be affected by the order of their entry. The proportional reduction in the rate of bad events in clinical trials suggested 52% relative risk reduction in analysis of patients number with satisfactory separation from parents, 22% relative risk reduction in analysis of patients number with satisfactory induction or mask acceptance, 34% relative risk reduction in analysis of patients number with requiring postoperative analgesics rescue, and 69% relative risk reduction in analysis of incidence of emergency agitation. And the TSA was performed by the use of Trial Sequential Analysis Viewer Software (version 0.9.5.10 beta; http://www.ctu.dk/tsa).

## 3 RESULTS

### 3.1 Literature search results

A total of 440 relevant items were identified initially. One hundred eighty‐four of them were excluded by duplicate removal, and 165 were excluded by reviewing the title and abstract. In these 165 excluded items, 69 were the protocols or registered trials (still recruiting or not), two were animal researches, 42 were studies performed in adult patients, 33 were unrelated reviews or meeting abstracts, 16 were studies with irrelevant topics, and three were similar systematic reviews published in 2014 and in 2015. A total of 57 items were excluded by full‐text reviewing, 54 of them were owing to the inappropriate comparisons, two of them reported the uncorrelated outcomes or the outcomes with inappropriate format, and the full text of the rest one cannot be gained after contacting the authors. Finally, 34 studies were selected in the consequent analysis.^14^, ^15^, ^16^, ^17^, ^18^, ^19^, ^20^, ^21^, ^22^, ^23^, ^24^, ^25^, ^26^, ^27^, ^28^, ^29^, ^30^, ^31^, ^32^, ^33^, ^34^, ^35^, ^36^, ^37^, ^38^, ^39^, ^40^, ^41^, ^42^, ^43^, ^44^, ^45^, ^46^, ^47^ The identification procedure of eligible items is described in Figure S1.

### 3.2 Basic characteristics of enrolled studies

These included studies were published from 2004 to 2019 (33 in English and one in Chinese) and were enrolled a total of 2281 pediatric patients (ages ranged from 6 months to 18 years). The primary outcomes “the number of patients with satisfactory separation from parents” and “the number of patients with satisfactory induction or mask acceptance” were reported separately in 18 studies and in 20 studies. And the primary adverse events “the incidence of postoperative pain needed analgesics rescue” and “the incidence of emergence agitation” were mentioned in eight studies and in 14 studies. The secondary outcomes including general hemodynamic parameters (systolic blood pressure, diastolic blood pressure, mean arterial pressure, and heart rate), onset of sedation, recovery time, and the incidences of various adverse events (shivering, bradycardia, nausea, and vomiting) were also reported in different studies. The main characteristics of these enrolled studies were summarized in Table 1.

### 3.3 Risk of bias assessment

In accordance with the Cochrane Collaboration tool for assessing risk of bias, we evaluated the mentioned‐above items. A total of 65% (22/34) studies performed an adequate method of random sequence generation, and 12 studies reported allocation concealment with detailed descriptions (using opaque, sealed envelopes). Twenty‐five studies described the blinding procedure of participants and personnel, and 25 studies mentioned the blinding procedure of outcome assessment. A total of eight studies were high‐quality studies with low risk of bias in all items. The detail of risk of bias assessment was shown in Figure S2.

### 3.4 Primary outcome 1: the number of patients with satisfactory separation from parents

**FIGURE 1:**
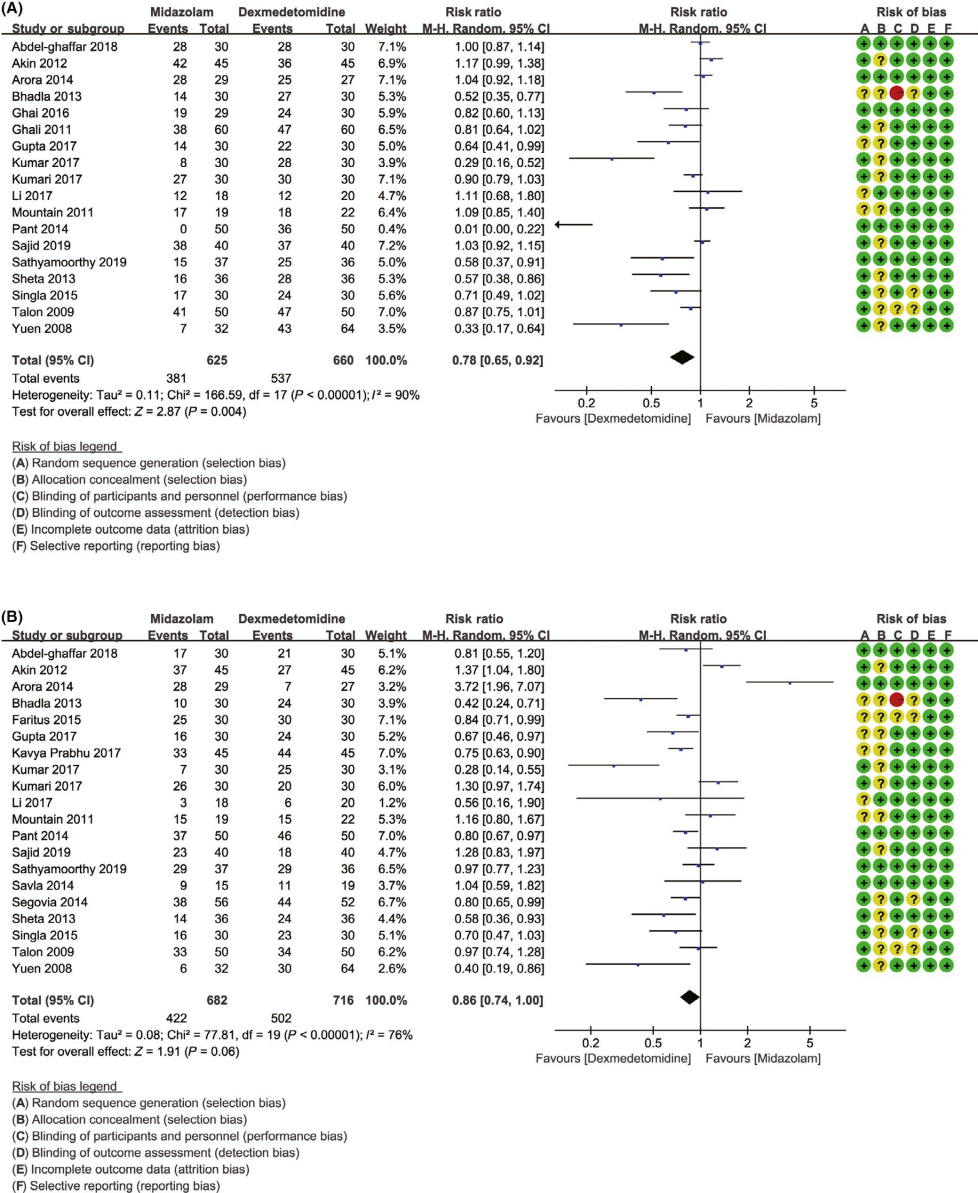
Effects of dexmedetomidine vs midazolam in number of patients with satisfactory separation from parents and in number of patients with satisfactory induction or mask acceptance. A, Forest plot depicting the meta‐analysis for the outcome “number of patients with satisfactory separation from parents”; B, Forest plot depicting the meta‐analysis for the outcome “number of patients with satisfactory induction or mask acceptance”

Eighteen studies with 1285 patients were enrolled.^17^, ^18^, ^20^, ^21^, ^23^, ^25^, ^26^, ^27^, ^28^, ^34^, ^36^, ^39^, ^41^, ^42^, ^43^, ^45^, ^46^, ^47^ The *I* ^2^ of 90% indicated substantial heterogeneity, but the source could not be clearly attributed to a single study by performing the sensitivity analysis; thus, the random effects model was used. The premedication of dexmedetomidine was associated with more satisfactory separation from parents compared to midazolam (81.36% vs 60.96%, RR = 0.78, with 95% CI [0.65, 0.92], *P = *.004, *I* ^2^ = 90%; Figure 1A). Although cumulative Z curves did not reach the required information size, the results of TSA indicated that the curves crossed both the conventional boundary and the trial sequential monitoring boundary. The level of evidence about the intervention effect was sufficient with unnecessary further trials (Figure 3A). Publication bias was detected in analysis by using of Begg's test (*P* = .006; Figure 4A). Therefore, in order to estimate and adjust for the number and outcomes of missing studies, we performed Duval's trim and fill method.^48^ And the results from sensitivity analyses of trim and fill method (no new studies added) revealed that the result was reliable.

### 3.5 Primary outcome 2: the number of patients with satisfactory induction or mask acceptance

A total of twenty studies^17^, ^18^, ^21^, ^23^, ^25^, ^26^, ^27^, ^28^, ^29^, ^30^, ^33^, ^34^, ^39^, ^40^, ^41^, ^42^, ^43^, ^45^, ^46^, ^47^ with 1398 patients were analyzed. The *I* ^2^ of 76% demonstrated that significant heterogeneity was existed. However, in sensitivity analysis, all attempts to reduce the value of *I* ^2^ to below 50% by excluding one single study were not successful. Therefore, random effects model was used. The using of dexmedetomidine was associated with higher rate of satisfactory induction or satisfactory mask acceptance compared to midazolam, but no significant differences were observed between two groups (71.11% vs 61.88%, RR = 0.86, with 95% CI [0.74, 1.00], *P* = .06, *I* ^2^ = 76%; Figure 1B). The TSA indicated that cumulative Z curves did not cross any of the boundaries, and the current number of patients was too few to achieve the required information size (2112 patients). The further evidences with large sample size are required (Figure 3B). Begg's (*P* = .381) test suggested that publication bias was not found (Figure 4B).

### 3.6 Primary outcome 3: the number of patients requiring postoperative analgesics rescue

**FIGURE 2:**
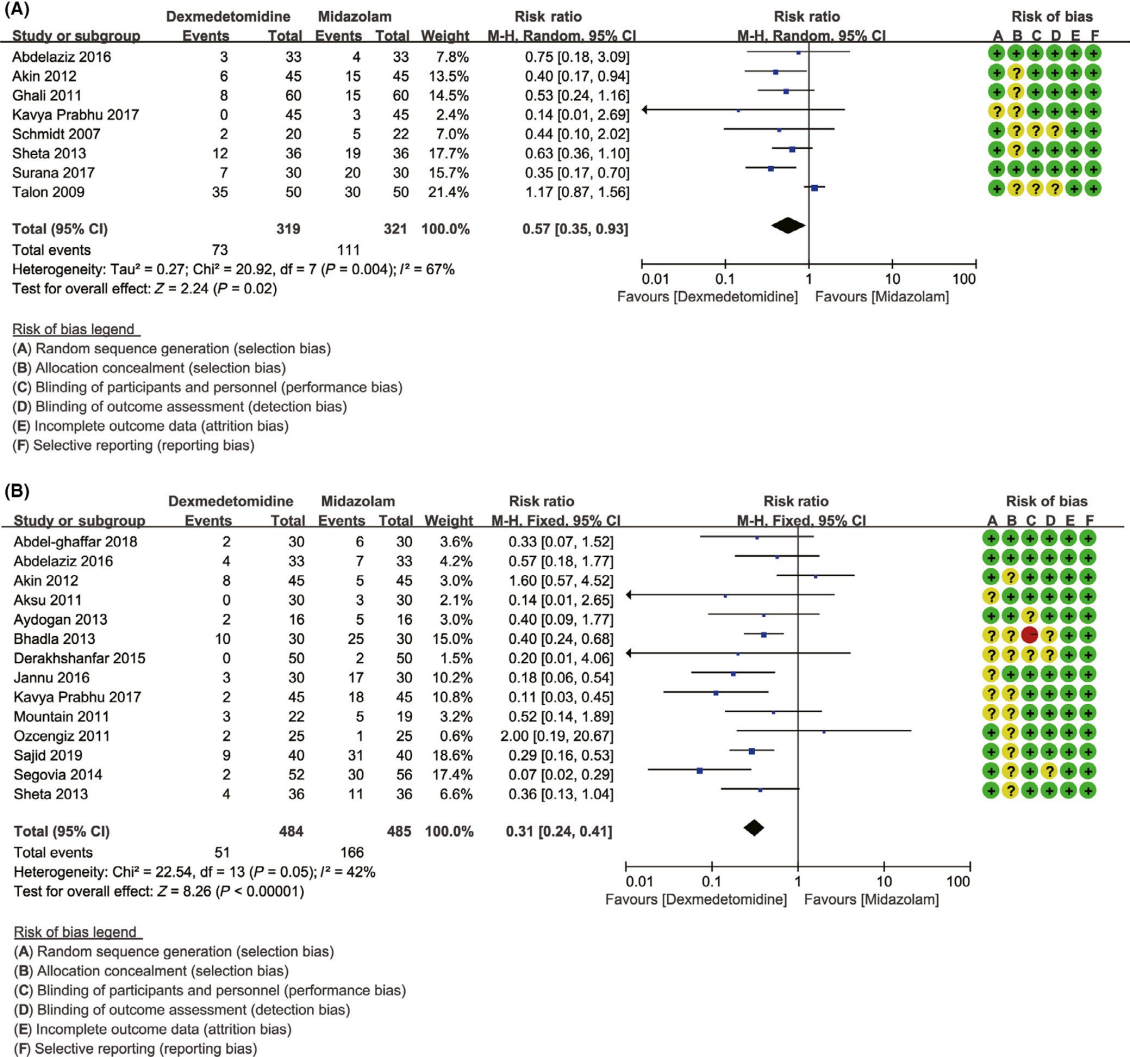
Effects of dexmedetomidine vs midazolam in number of patients requiring postoperative analgesics rescue and in incidence of emergence agitation. A, Forest plot depicting the meta‐analysis for the outcome “number of patients requiring postoperative analgesics rescue”; B, Forest plot depicting the meta‐analysis for the outcome “incidence of emergence agitation”

**FIGURE 3:**
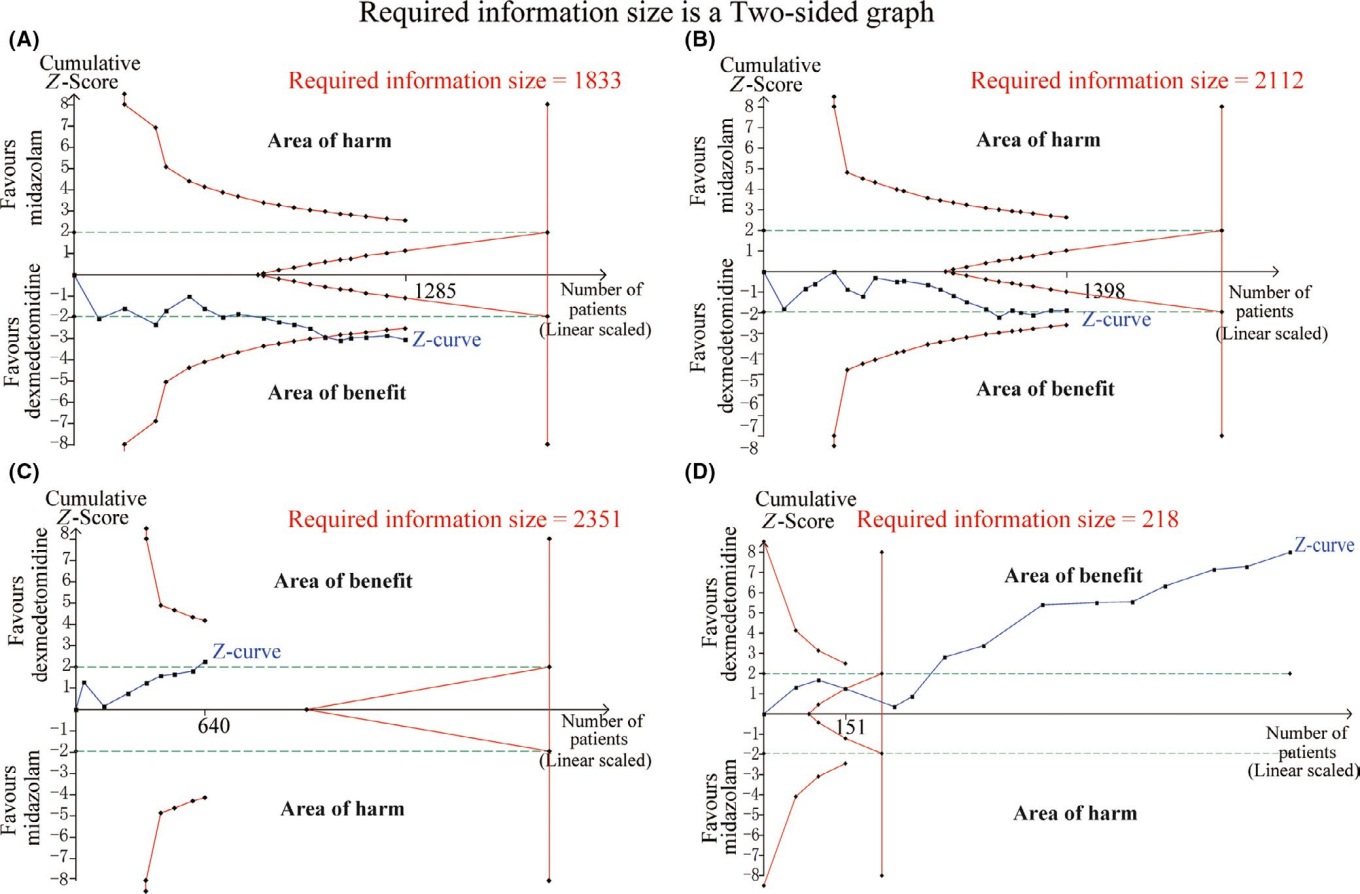
A, Trial sequential analysis for the outcome “number of patients with satisfactory separation from parents.” B, Trial sequential analysis for the outcome “number of patients with satisfactory induction or mask acceptance.” C, Trial sequential analysis for the outcome “number of patients requiring postoperative analgesics rescue.” D, Trial sequential analysis for the outcome “incidence of emergence agitation.”

**FIGURE 4:**
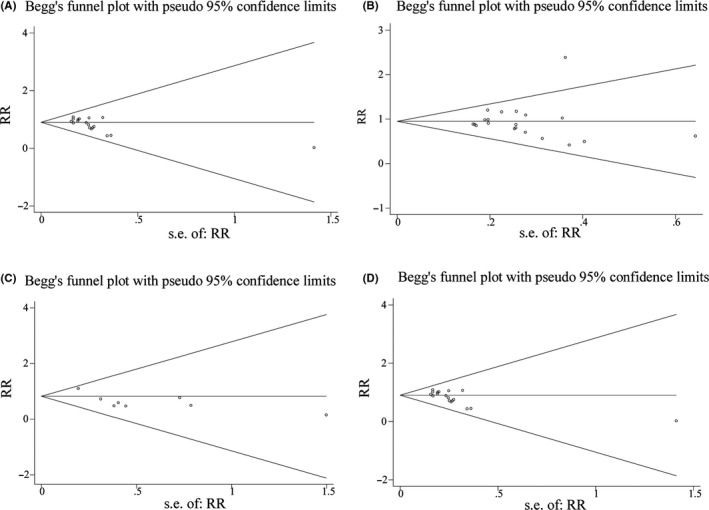
Funnel plots of effect estimates for the co‐primary outcomes. A, the number of patients with satisfactory separation from parents; B, the number of patients with satisfactory induction or mask acceptance; C, the number of patients requiring postoperative analgesics rescue; D, the incidence of emergence agitation. RR, risk ratio; SE, standard error

It was reported in eight studies with 640 patients.^16^, ^20^, ^23^, ^26^, ^28^, ^35^, ^40^, ^44^ Patients who received dexmedetomidine experienced significantly lower incidence of postoperative analgesics rescue than patients who received midazolam (22.88% vs 34.58%, RR = 0.57, with 95% CI [0.35, 0.93], *P* = .02; *I* ^2^ = 67% (Figure 2A). The sensitivity analysis indicated that the substantial heterogeneity (*I* ^2^ = 67%) was attributable to the Talon et al study.^18^ Heterogeneity was resolved (*I* ^2^ = 0%) by removing this study, and the summary estimate was unchanged essentially (14.13% vs 29.89%, RR = 0.47, 95% CI [0.34, 0.66], *P* < .00001).The TSA showed that cumulative Z curves crossed the conventional boundary for benefit but did not cross both trial sequential monitoring boundary and required information size. It might reveal a possible false‐positive effect of dexmedetomidine in reducing the incidence of postoperative severe pain compared to midazolam. The further trials to achieve the firm evidences are necessary (Figure 3C). Begg's test (*P* = .711) indicated that publication bias was not found in the analysis (Figure 4C).

### 3.7 Primary outcome 4: the incidence of emergence agitation

The emergence agitation was mentioned in 14 studies with 969 patients.^19^, ^21^, ^22^, ^23^, ^24^, ^25^, ^26^, ^30^, ^32^, ^35^, ^38^, ^40^, ^45^, ^46^ Emergence agitation was significantly infrequent in patients in dexmedetomidine group compared with the midazolam group (10.54% vs 34.23%, RR = 0.31, with 95% CI [0.24, 0.41], *P < *.00001, *I* ^2^ = 42%; Figure 2B). Given that the value of *I* ^2^ was 42%, the fixed‐effects model was used. The outcome of TSA demonstrated that the cumulative Z curves crossed the conventional boundary, trial sequential monitoring boundary, and the required information size (calculated as 218). It suggested that the answer of such clinical question was definitively clear and the sample size of patients was enough. Further studies are unlikely to change the conclusions and are unnecessary (Figure 3D). After Begg's test (*P* = .827), no publication bias was found in the analysis (Figure 4D).

### 3.8 Secondary outcomes

All secondary outcomes involving hemodynamic parameters, onset of sedation, recovery time, and incidence of different adverse effects were clarified in Table 2. The details about general hemodynamic parameters including systolic blood pressure (SBP), diastolic blood pressure (DBP), mean arterial pressure (MAP), and heart rate (HR) were reported separately in six studies,^14^, ^20^, ^25^, ^31^, ^33^, ^34^ three studies,^14^, ^31^, ^33^ two studies,^15^, ^16^ and eight studies.^14^, ^15^, ^16^, ^20^, ^25^, ^31^, ^33^, ^34^ The results indicated that the using of dexmedetomidine was associated with significant less value of SBP (SMD = 0.99, with 95% CI [0.19, 1.78], *P* = .02; *I* ^2^ = 91%) and heart rate (SMD = 1.27, with 95% CI [0.61, 1.94], *P* = .0002; *I* ^2^ = 90%) in pediatric patients.

**Table 2:** Secondary outcomes

Outcomes	Number of studies (Reference no.)	Number of dexmedetomidine patients	Number of midazolam patients	*I* ^2^ (%)	Standardized mean difference with [95% CI]	*P* value
Systolic blood pressure (SBP)	6 (14, 20, 25, 31, 33, 34)	181	181	91	0.99 [0.19, 1.78]	.02*
Diastolic blood pressure (DBP)	3 (14, 31, 33)	61	61	0	0.17 [−0.19, 0.52]	.36
Mean arterial pressure (MAP)	2 (15, 16)	80	62	0	−0.26 [−0.59, 0.08]	.14
Heart rate (HR)	8 (14, 15, 16, 20, 25, 31, 33)	241	243	90	1.27 [0.61, 1.94]	.0002*
Onset of sedation	6 (15, 26, 31, 37, 38, 43)	178	175	98	−0.26 [−2.04, 1.52]	.78
Recovery time	6 (15, 16, 19, 20, 26, 31, 40)	231	233	63	−0.10 [−0.41, 0.21]	.51

Outcomes	Number of studies (Reference no.)	Dexmedetomidine patients (Incidence, %)	Midazolam patients (Incidence, %)	*I* ^2^ (%)	Risk ratio with [95% CI]	*P* value
Incidence of Bradycardia	4 (24, 37, 44, 46)	12/117 (10.26%)	1/116 (0.86%)	0	5.37 [1.44, 20.02]	.01*
Incidence of Nauseas/vomiting	4 (23, 26, 35, 45)	20/144 (13.89%)	25/144 (17.36%)	0	0.80 [0.48, 1.36]	.41
Incidence of Shivering	2 (25, 26)	11/66 (16.67%)	28/66 (42.42%)	0	0.39 [0.22, 0.70]	.002*

* Significant difference between groups (*P* < .05).

The difference of onset of sedation between two groups was not significant (SMD = −0.26, with 95% CI [−2.04, 1.52], *P* = .78, *I* ^2^ = 98%), which was inconsistent with the previous outcome from the similar systematic review.^9^ It might be resulted from the different sample sizes. And we also found two groups did not differ significantly in recovery time.

The analysis of various adverse events exhibited that the patients in dexmedetomidine group suffered increased incidence of bradycardia, and experienced lower rate of shivering. The incidence of postoperative nauseas or vomiting was not different between two groups. However, the reports about the incidence of the adverse events were relatively scarce.

### 3.9 Quality of the evidence

We used GRADE approach to grading the level of each outcome in present study. Although the results from risk of bias assessment part indicated that the quality of trials design was reasonable, the GRADE summary of findings table demonstrated that the overall level of current evidence in our meta‐analysis was moderate or low, which might be resulted from the inconsistency issue and, particularly, the limited number of events (Table S1).

## 4 DISCUSSION

Pediatric sedation is always served as one of conundrums during diagnostic and surgical procedures, such area changes rapid and engenders several debates among the anesthesiologists and pediatric specialists.^49^ For instance, the optimal premedication between dexmedetomidine and midazolam for pediatric sedation also remains controversial. Although relevant meta‐analyses published during 2014‐2015^8^, ^9^ seemed to validate the superiority of dexmedetomidine both in providing sedative effects and in alleviating adverse events compared to midazolam, the limited sample size and the recent published studies with inconsistent conclusions prompted us to update the analysis (eg, Abdel‐Ghaffar et al^45^ suggested that no significant differences were found in sedative level at parental separation between two groups; Sajid et al^46^ and Sathyamoorthy et al^47^ showed that sedative level at mask induction of children in two groups was approximately similar). Compared with the previous meta‐analyses, we added latest evidences from the literature on their efficacy and safety in pediatric sedation to evaluate comprehensively. In addition, we performed trial sequential analysis to investigate whether these primary outcomes achieved the required information size to draw the conclusions. And the Grading of Recommendations Assessment, Development, and Evaluation (GRADE) methodology was used to assess the quality of the current evidences for reference.

The analysis of co‐primary outcomes indicated that patients received dexmedetomidine as premedication were associated with less incidence of emergence agitation and postoperative pain. The results strengthened the previous findings and illustrated the potential analgesic effects from alpha‐2 agonist.^50^ The results from TSA about incidence of emergence agitation between two groups indicated that the current sample size went beyond the required information size and the present evidences of anticipated intervention effects were sufficient. Thereby the similar further studies are unnecessary to be performed. However, possible false‐positive effect might be exhibited in dexmedetomidine group in decreasing the incidence of postoperative severe pain, since the cumulative Z curves in TSA only cross the conventional boundary for benefit.

The analysis about number of patients with satisfactory parent separation following premedication with two drugs suggested that dexmedetomidine was more effective than midazolam in alleviating children's anxiety at separation from parents. Compared with previous similar studies, the TSA indicated that level of evidence about the intervention effect from dexmedetomidine might be sufficient and reliable with unnecessary further trials. However, the present analysis, by accumulating the new evidences, did not verify the superiority of dexmedetomidine in producing satisfactory sedation at mask induction.

Our study updated the previous conclusion about the superiority of dexmedetomidine in onset of sedation.^9^ The combination of studies with expanded sample size demonstrated that the difference between dexmedetomidine and midazolam in onset time was not significant. Moreover, the summary of new evidences also indicated that no difference was found in recovery time between two groups.

Noteworthily, in evaluation of hemodynamic parameters and adverse events between two groups, the using of dexmedetomidine exhibited great reduction in systolic blood pressure and heart rate, and was associated with high incidence of bradycardia. These phenomena might be derived from the biphasic effects of α2‐adrenoceptor. It enhanced the blood pressure temporarily as the transient vasoconstrictive effects in peripheral vasculature and then lowered the arterial pressure with decreasing sympathetic outflow.^51^ Even though, some researchers still regarded the dexmedetomidine as one appropriate sedative option for pediatric patients, in consideration of great hemodynamic changes could be resolved by decelerating the rate of administration.^52^, ^53^

The secondary outcomes about different adverse events suggested that incidence of shivering was lower in patients received dexmedetomidine compared to midazolam. And no difference was found in occurrence rate of postoperative nausea and vomiting between two groups. However, owing to extremely limited sample size, the above data were not enough to draw a definitive and reliable conclusion. This was one of the limitations in present study. Hence, the focus in future should be moved on the evaluation of safety in using dexmedetomidine and midazolam as premedication in children.

Furthermore, the widespread moderate or low quality in outcomes evaluated by GRADE approach resulted from inconsistency (high heterogeneity) and imprecision (lack of events number). Heterogeneity might be originated from different types of procedures, administration routes, and premedication doses. The sensitivity analysis performed by us discovered one trial which brought the significant heterogeneity in evaluation of patients requiring postoperative analgesics rescue, and then, we verified the reliability of conclusion by omitting it. And the other significant heterogeneity among studies led us to use random effects models for meta‐analysis.

## 5 CONCLUSION

In conclusion, the current evidences suggest that dexmedetomidine is the preferred choice for pediatric patients than midazolam owing to its more satisfactory sedation at separation from parents and less incidence of emergence agitation. However, the superiority of using dexmedetomidine as premedication in providing adequate sedation at mask induction and postoperative analgesic effects compared to midazolam has not yet been defined. Additionally, to obtain firm evidences about the effects of dexmedetomidine vs midazolam on hemodynamic parameters and the safety of two premedicants, more high‐quality trials are required. And the exploration of the optimal dose range and ideal route of using dexmedetomidine should also be considered in future.

## CONFLICT OF INTEREST

The authors declare no conflict of interest.

## Supporting information

Appendix S1

Figure S1

Figure S2

Table S1

Supplementary Material
