# Corrigendum

**DOI:** 10.1111/cns.13986

**Published:** 2022-11-11

A comparative evaluation of dexmedetomidine and midazolam in pediatric sedation: A meta‐analysis of randomized controlled trials with trial sequential analysis, published on April 29, 2020 https://doi.org/10.1111/cns.13377.

(1) In present research, the authors analyzed four primary outcomes including the number of patients with satisfactory separation from parents, the number of patients with satisfactory induction or mask acceptance, the number of patients requiring postoperative analgesics rescue, and the incidence of emergence agitation to evaluate the efficacy and safety of dexmedetomidine and midazolam in pediatric sedation. In addition, the authors evaluated the publication bias of outcomes and presented funnel plots (Figure 1) (Page 872 of the original text, Figure 4 A‐D).

**FIGURE 1:**
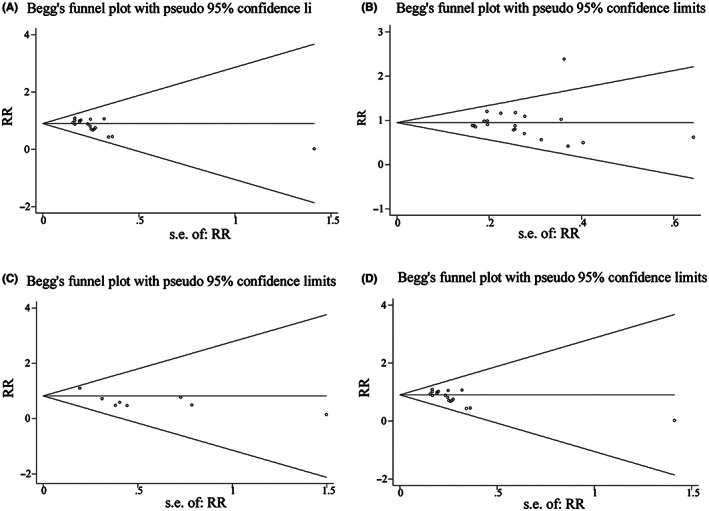
Figure 4 A‐D in original manuscript

Recently, authors found that they repeatedly placed Figure 4A (funnel plot of outcome “number of patients with satisfactory separation from parents”) in the position of Figure 4D (funnel plot of outcome “incidence of emergence agitation”) when merging four pictures. It resulted in repeated appearance of Figure 4A and the unsuccessful upload of Figure 4D. The correct Figure 4D is given below (Figure 2):

**FIGURE 2:**
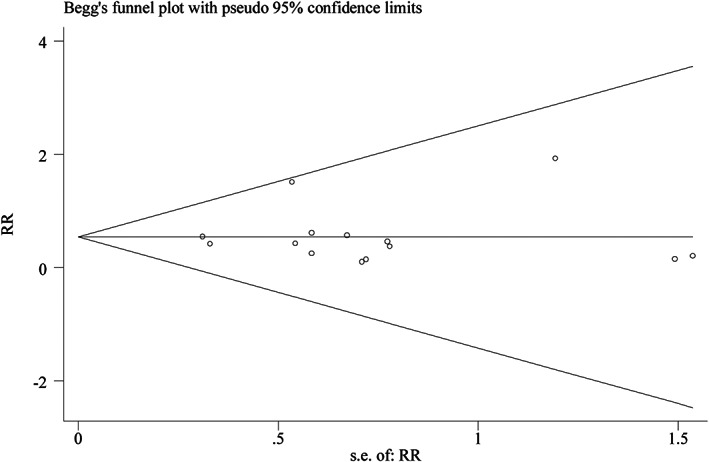
The correct Figure 4D

According to verification, it is actually caused by the author's negligence in uploading process. However, the results and conclusions in original article are derived from the correct funnel plot of outcome “incidence of emergence agitation”. The results and conclusions of the original article are not affected.

(2) In abstract section (Page 862, Line 14) “The results indicated that administration of dexmedetomidine was associated with less incidence of emergence agitation (RR = 0.78, with 95% CI [0.65, 0.92]) and more satisfactory sedation at parental separation (RR = 0.31, with 95% CI [0.24, 0.41]) compared to midazolam” should be “The results indicated that administration of dexmedetomidine was associated with more satisfactory sedation at parental separation (RR = 0.78, with 95% CI [0.65, 0.92]) and less incidence of emergence agitation (RR = 0.31, with 95% CI [0.24, 0.41]) compared to midazolam”.

According to verification, it is incorrect sequence of outcome description caused by the author's negligent typing. However, the data, results and conclusions in original article are derived from the correct forest plots, and the results and conclusions of the original article are not affected.

Authors apply for correction Figure 4D and the description of abstract. The authors apologize for the above mentioned errors.

