# Supplementary material for: A comparative evaluation of dexmedetomidine and midazolam in pediatric sedation: A meta‐analysis of randomized controlled trials with trial sequential analysis
Source: CNS Neurosci Ther. 2020 Apr 29;26(8):862–75. doi: 10.1111/cns.13377 (PMC7366749; doi:10.1111/cns.13377)
Supplement: Supplementary file 1 — Appendix S1 [file CNS-26-862-s001.docx]

2019.10.7

**Pubmed**

| **Search** | **Query** | **Items found** |
| --- | --- | --- |
| #1 | Infant [mesh] | 1108962 |
| #2 | newborn* [tiab] or neonat* [tiab] or infant* [tiab] or infancy [tiab] or baby [tiab] or babies [tiab] or toddler* [tiab] | 765100 |
| #3 | #1 OR #2 | 1409272 |
| #4 | Child [mesh] | 1852528 |
| #5 | Pediatrics [mesh] | 56139 |
| #6 | p?ediatric* [tiab] or child* [tiab] or kindergar* [tiab] or preschool* [tiab] or kid [tiab] or kids [tiab] or schoolchild* [tiab] or “school age” [tiab] or schoolage [tiab] or preteen* [tiab] or youth* [tiab] or prepubescent* [tiab] | 1428480 |
| #7 | #4 OR #5 OR #6 | 2376511 |
| #8 | Adolescent [mesh] | 1960562 |
| #9 | adolesc* [tiab] or teen* [tiab] or youth* [tiab] or underage* [tiab] or “under age*” [tiab] or minor* [tiab] or juvenile* [tiab] or pubert* [tiab] or pubescen* [tiab] or “young people*” [tiab] or “young person*” [tiab] or “young adult*” [tiab] | 757633 |
| #10 | #8 OR #9 | 2406105 |
| #11 | #3 OR #7 OR #10 | 4368253 |
| #12 | midazolam [tiab] OR midazolamum [tiab] OR buccolam [tiab] OR seizalam [tiab] OR Anquil [tiab] OR Benzosed [tiab] OR Dalam [tiab] OR Damizol [tiab] OR Demizolam [tiab] OR Doricum [tiab] OR Dormicum [tiab] OR Dormid [tiab] OR Dormipron [tiab] OR Dormire [tiab] OR Dormitol [tiab] OR Dormixal [tiab] OR Dormonid [tiab] OR Drimnorth [tiab] OR Epistatus [tiab] OR Flormida [tiab] OR Fulsed [tiab] OR Garen [tiab] OR Gobbizolam [tiab] OR Hipnazolam [tiab] OR Hipnoz [tiab] OR Hypnofast [tiab] OR Hypnovel [tiab] OR Ipnovel [tiab] OR Nocturna [tiab] OR Setam [tiab] OR Talentum [tiab] OR Terap [tiab] OR Versed [tiab] | 13882 |
| #13 | dexmedetomidin* [tiab] OR Precedex [tiab] OR Dexdor [tiab] OR Dexem [tiab] OR DEX [tiab] | 13901 |
| #14 | (randomized controlled trial [pt] OR controlled clinical trial [pt] OR randomized [tiab] OR placebo [tiab] OR clinical trials as topic [mesh: noexp] OR randomly [tiab] OR trial [ti]) NOT (animals [mh] NOT humans [mh]) | 1151229 |
| #15 | #11 AND #12 AND #13 AND #14 | 105 |

**Embase**

| **Search** | **Query** | **Items found** |
| --- | --- | --- |
| #1 | 'Infant'/exp | 1200076 |
| #2 | (newborn* or neonat* or infant* or infancy or baby or babies or toddler*):ab,ti | 908563 |
| #3 | #1 OR #2 | 1,491,681 |
| #4 | 'child'/exp | 2,782,246 |
| #5 | 'pediatrics'/exp | 112,174 |
| #6 | (paediatric*or pediatric* or child* or kindergar* or preschool* or kid or kids or schoolchild* or 'school age' or schoolage or preteen* or youth* or prepubescent*):ab,ti | 1,810,037 |
| #7 | #4 OR #5 OR #6 | 3,353,107 |
| #8 | 'adolescent'/exp | 1,605,148 |
| #9 | (adolesc* or teen* or youth* or underage* or "under age*" or minor* or juvenile* or pubert* or pubescen* or "young people*" or "young person*" or "young adult*"):ab,ti | 1,013,427 |
| #10 | #8 or #9 | 2,255,485 |
| #11 | (#3 or #7 or #10) and [embase]/lim | 3,220,347 |
| #12 | (midazolam or midazolamum or buccolam or seizalam or Anquil or Benzosed or Dalam or Damizol or Demizolam or Doricum or Dormicum or Dormid or Dormipron or Dormire or Dormitol or Dormixal or Dormonid or Drimnorth or Epistatus or Flormida or Fulsed or Garen or Gobbizolam or Hipnazolam or Hipnoz or Hypnofast or Hypnovel or Ipnovel or Nocturna or Setam or Talentum or Terap or Versed):ab,ti | 20,797 |
| #13 | #12 and [embase]/lim | 18,673 |
| #14 | (dexmedetomidin* OR Precedex OR Dexdor OR Dexem OR DEX):ab,ti | 19,662 |
| #15 | #14 and [embase]/lim | 17,910 |
| #16 | 'randomized controlled trial'/exp | 571,170 |
| #17 | 'controlled clinical trial'/exp | 740,162 |
| #18 | 'randomization'/exp | 84,211 |
| #19 | 'double blind procedure'/exp | 165,576 |
| #20 | 'single blind procedure'/exp | 36,516 |
| #21 | random*:ab | 1,414,663 |
| #22 | trial*:ab | 1,233,816 |
| #23 | #17 OR #18 OR #19 OR #20 OR #21 OR #22 | 2,386,129 |
| #24 | 'human'/exp | 21,175,426 |
| #25 | #23 AND #24 AND [embase]/lim | 1,660,726 |
| #26 | #11 AND #13 AND #15 AND #25 | 116 |

**Cochrane Library**

| **Search** | **Query** | **Items found** |
| --- | --- | --- |
| #1 | MeSH descriptor: [Infant] explode all trees | 15567 |
| #2 | (newborn* or neonat* or infant* or infancy or baby or babies or toddler*):ti,ab,kw | 71792 |
| #3 | #1 or #2 | 71792 |
| #4 | MeSH descriptor: [Child] explode all trees | 1207 |
| #5 | MeSH descriptor: [Pediatrics] explode all trees | 644 |
| #6 | (paediatric*or pediatric* or child* or kindergar* or preschool* or kid or kids or schoolchild* or 'school age' or schoolage or preteen* or youth* or prepubescent*):ti,ab,kw | 151240 |
| #7 | #4 or #5 or #6 | 151414 |
| #8 | MeSH descriptor: [Adolescent] explode all trees | 101087 |
| #9 | (adolesc* or teen* or youth* or underage* or "under age*" or minor* or juvenile* or pubert* or pubescen* or "young people*" or "young person*" or "young adult*"):ti,ab,kw | 194365 |
| #10 | #8 or #9 | 194365 |
| #11 | #3 or #7 or #10 | 326042 |
| #12 | (midazolam or midazolamum or buccolam or seizalam or Anquil or Benzosed or Dalam or Damizol or Demizolam or Doricum or Dormicum or Dormid or Dormipron or Dormire or Dormitol or Dormixal or Dormonid or Drimnorth or Epistatus or Flormida or Fulsed or Garen or Gobbizolam or Hipnazolam or Hipnoz or Hypnofast or Hypnovel or Ipnovel or Nocturna or Setam or Talentum or Terap or Versed):ti,ab,kw | 10301 |
| #13 | (dexmedetomidin* OR Precedex OR Dexdor OR Dexem OR DEX):ti,ab,kw | 5338 |
| #14 | #11 and #12 and #13 | 223  (4 reviews; 219 Trials) |
