# Supplementary figures and images for: A comparative evaluation of dexmedetomidine and midazolam in pediatric sedation: A meta‐analysis of randomized controlled trials with trial sequential analysis
Source: CNS Neurosci Ther. 2020 Apr 29;26(8):862–75. doi: 10.1111/cns.13377 (PMC7366749; doi:10.1111/cns.13377)

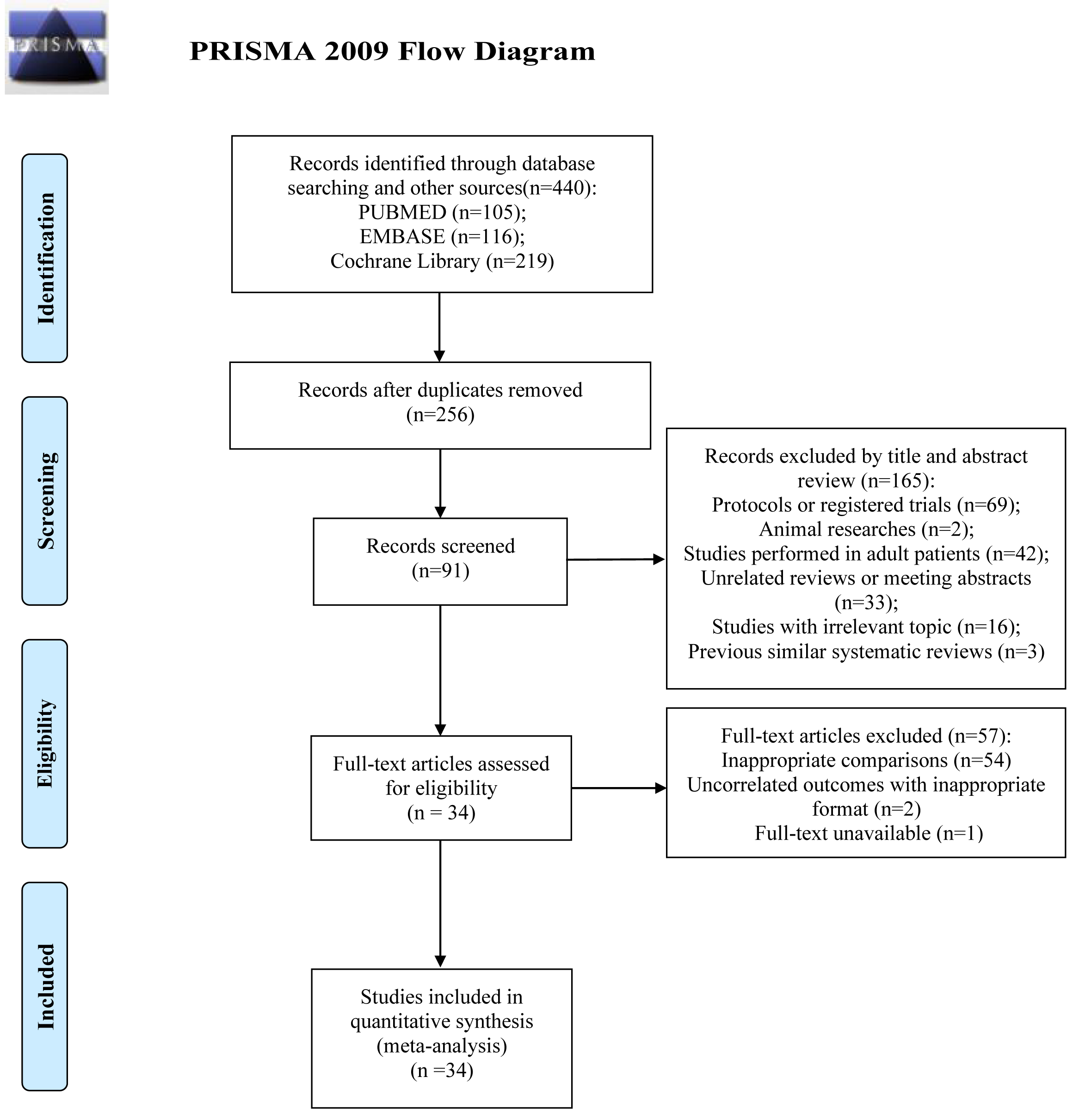

Supplement: Supplementary file 2 — Figure S1 [file CNS-26-862-s002.tiff]

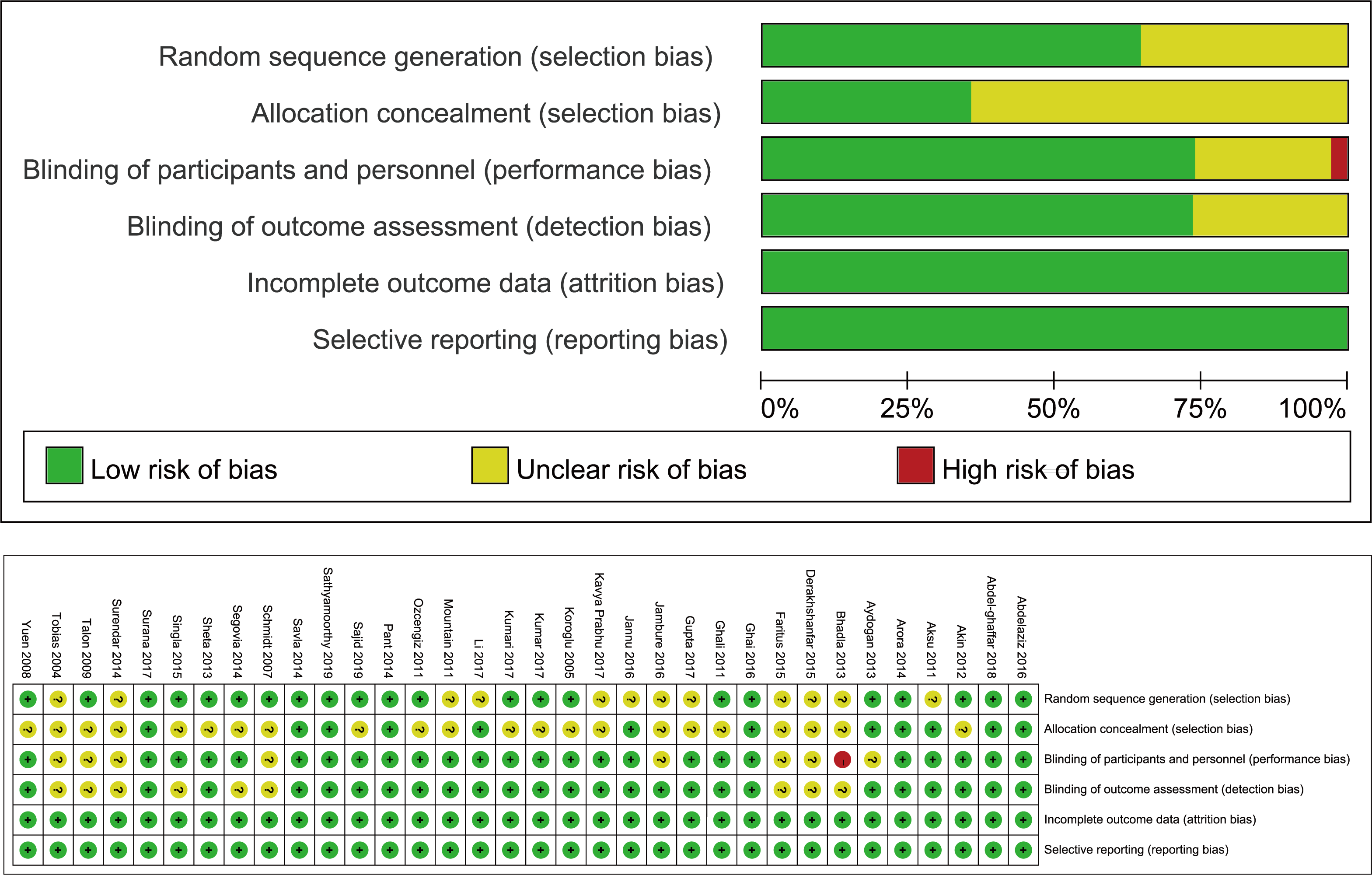

Supplement: Supplementary file 3 — Figure S2 [file CNS-26-862-s003.tif]
