# Supplementary material for: A comparative evaluation of dexmedetomidine and midazolam in pediatric sedation: A meta‐analysis of randomized controlled trials with trial sequential analysis
Source: CNS Neurosci Ther. 2020 Apr 29;26(8):862–75. doi: 10.1111/cns.13377 (PMC7366749; doi:10.1111/cns.13377)
Supplement: Supplementary file 4 — Table S1 [file CNS-26-862-s004.docx]

**Table S1. GRADE summary of findings table**

| **Quality assessment** | | | | | | | **Summary of Findings** | | | | |
| --- | --- | --- | --- | --- | --- | --- | --- | --- | --- | --- | --- |
| **Participants (studies)**  **Follow up** | **Risk of bias** | **Inconsistency** | **Indirectness** | **Imprecision** | **Publication bias** | **Overall quality of evidence** | **Study event rates (%)** | | **Relative effect** (95% CI) | **Anticipated absolute effects** | |
|  |  |  |  |  |  |  | **With Control** | **With Number of patients with satisfactory separation from parents** |  | **Risk with Control** | **Risk difference with Number of patients with satisfactory separation from parents** (95% CI) |
| **Primary outcomes** | | | | | | | | | | | |
| **Number of patients with satisfactory separation from parents (CRITICAL OUTCOME)** | | | | | | | | | | | |
| 1285  (18 studies) | no serious risk of bias | serious^1^ | no serious indirectness | no serious imprecision | reporting bias^2^ | **⊕⊕OO LOW** | 537/660  (81.4%) | 381/625  (61%) | RR 0.78  (0.65 to 0.92) | **Study population** | |
|  |  |  |  |  |  |  |  |  |  | 814 per 1000 | 179 fewer per 1000 (from 65 fewer to 285 fewer) |
|  |  |  |  |  |  |  |  |  |  | **Moderate** | |
|  |  |  |  |  |  |  |  |  |  | 800 per 1000 | 176 fewer per 1000 (from 64 fewer to 280 fewer) |
| **Number of patients with satisfactory induction or mask acceptance (CRITICAL OUTCOME)** | | | | | | | | | | | |
| 1398  (20 studies) | no serious risk of bias | serious^1^ | no serious indirectness | no serious imprecision | none | **⊕⊕⊕O MODERATE** | 502/716  (70.1%) | 422/682  (61.9%) | RR 0.86  (0.74 to 1) | **Study population** | |
|  |  |  |  |  |  |  |  |  |  | 701 per 1000 | 98 fewer per 1000 (from 182 fewer to 0 more) |
|  |  |  |  |  |  |  |  |  |  | **Moderate** | |
|  |  |  |  |  |  |  |  |  |  | 691 per 1000 | 97 fewer per 1000 (from 180 fewer to 0 more) |
| **The incidence of emergence agitation (CRITICAL OUTCOME)** | | | | | | | | | | | |
| 969  (14 studies) | no serious risk of bias | no serious inconsistency | no serious indirectness | serious^3^ | none | **⊕⊕⊕O MODERATE** | 51/484 (10.5%) | 166/485  (34.2%) | RR 0.31  (0.24 to 0.41) | **Study population** | |
|  |  |  |  |  |  |  |  |  |  | 342 per 1000 | 236 fewer per 1000 (from 202 fewer to 260 fewer) |
|  |  |  |  |  |  |  |  |  |  | **Moderate** | |
|  |  |  |  |  |  |  |  |  |  | 284 per 1000 | 196 fewer per 1000 (from 168 fewer to 216 fewer) |
| **Number of patients requiring postoperative analgesia rescue (CRITICAL OUTCOME)** | | | | | | | | | | | |
| 640  (8 studies) | no serious risk of bias | serious^1^ | no serious indirectness | serious^3^ | none | **⊕⊕OO LOW** | 73/319 (22.9%) | 111/321  (34.6%) | RR 0.57  (0.35 to 0.93) | **Study population** | |
|  |  |  |  |  |  |  |  |  |  | 346 per 1000 | 149 fewer per 1000 (from 24 fewer to 225 fewer) |
|  |  |  |  |  |  |  |  |  |  | **Moderate** | |
|  |  |  |  |  |  |  |  |  |  | 292 per 1000 | 126 fewer per 1000 (from 20 fewer to 190 fewer) |
| **Secondary outcomes** | | | | | | | | | | | |
| **Hemodynamic status(SBP) (IMPORTANT OUTCOME)** | | | | | | | | | | | |
| 362  (6 studies) | no serious risk of bias | serious^1^ | no serious indirectness | serious^4^ | none | **⊕⊕OO LOW** | 181 | 181 | - | SMD 0.99 higher (0.19 to 1.78 higher) | |
| **Hemodynamic status(DBP) (IMPORTANT OUTCOME)** | | | | | | | | | | | |
| 122  (3 studies) | no serious risk of bias | no serious inconsistency | no serious indirectness | serious^4^ | none | **⊕⊕⊕O**  **MODERATE** | 61 | 61 | - | SMD 0.17 higher (0.19 lower to 0.52 higher) | |
| **Hemodynamic status(MAP) (IMPORTANT OUTCOME)** | | | | | | | | | | | |
| 142  (2 studies) | no serious risk of bias | no serious inconsistency | no serious indirectness | serious^4^ | none | **⊕⊕⊕O MODERATE** | 80 | 62 | - | SMD 0.26 lower (0.59 lower to 0.08 higher) | |
| **Hemodynamic status(HR) (IMPORTANT OUTCOME)** | | | | | | | | | | | |
| 484  (8 studies) | no serious risk of bias | serious^1^ | no serious indirectness | no serious imprecision | none | **⊕⊕⊕O MODERATE** | 241 | 243 | - | SMD 1.27 higher (0.61 to 1.94 higher) | |
| **Onset of sedation (IMPORTANT OUTCOME)** | | | | | | | | | | | |
| 353  (6 studies) | no serious risk of bias | serious^1^ | no serious indirectness | serious^4^ | none | **⊕⊕OO LOW** | 175 | 178 | - | SMD 0.26 lower (2.04 lower to 1.52 higher) | |
| **Recovery time (IMPORTANT OUTCOME)** | | | | | | | | | | | |
| 353  (6 studies) | no serious risk of bias | serious^1^ | no serious indirectness | serious^4^ | none | **⊕⊕OO LOW** | 175 | 178 | - | SMD 0.26 lower (2.04 lower to 1.52 higher) | |
| **Onset of sedation (IMPORTANT OUTCOME)** | | | | | | | | | | | |
| 353  (6 studies) | no serious risk of bias | serious^1^ | no serious indirectness | serious^4^ | none | **⊕⊕OO LOW** | 175 | 178 | - | SMD 0.26 lower (2.04 lower to 1.52 higher) | |
| **Recovery time (IMPORTANT OUTCOME)** | | | | | | | | | | | |
| 464  (6 studies) | no serious risk of bias | serious^1^ | no serious indirectness | no serious imprecision | none | **⊕⊕⊕O MODERATE** | 231 | 233 | - | SMD 0.1 lower (0.41 lower to 0.21 higher) | |
| **The incidence of Bradycardia (IMPORTANT OUTCOME)** | | | | | | | | | | | |
| 233  (4 studies) | no serious risk of bias | no serious inconsistency | no serious indirectness | very serious^5^ | none | **⊕⊕OO LOW** | 12/117 (10.3%) | 1/116  (0.9%) | RR 5.37  (1.44 to 20.02) | **Study population** | |
|  |  |  |  |  |  |  |  |  |  | 9 per 1000 | 38 more per 1000 (from 4 more to 164 more) |
|  |  |  |  |  |  |  |  |  |  | **Moderate** | |
|  |  |  |  |  |  |  |  |  |  | - | - |
| **The incidence of Nauseas** **and vomiting (IMPORTANT OUTCOME)** | | | | | | | | | | | |
| 288  (4 studies) | no serious risk of bias | no serious inconsistency | no serious indirectness | very serious^5^ | none | **⊕⊕OO LOW** | 20/144 (13.9%) | 25/144  (17.4%) | RR 0.8  (0.48 to 1.36) | **Study population** | |
|  |  |  |  |  |  |  |  |  |  | 174 per 1000 | 35 fewer per 1000 (from 90 fewer to 62 more) |
|  |  |  |  |  |  |  |  |  |  | **Moderate** | |
|  |  |  |  |  |  |  |  |  |  | 176 per 1000 | 35 fewer per 1000 (from 92 fewer to 63 more) |
| **The incidence of Shivering (IMPORTANT OUTCOME)** | | | | | | | | | | | |
| 132  (2 studies) | no serious risk of bias | no serious inconsistency | no serious indirectness | very serious^5^ | none | **⊕⊕OO LOW** | 11/66 (16.7%) | 28/66  (42.4%) | RR 0.39  (0.22 to 0.7) | **Study population** | |
|  |  |  |  |  |  |  |  |  |  | 424 per 1000 | 259 fewer per 1000 (from 127 fewer to 331 fewer) |
|  |  |  |  |  |  |  |  |  |  | **Moderate** | |
|  |  |  |  |  |  |  |  |  |  | 442 per 1000 | 270 fewer per 1000 (from 133 fewer to 345 fewer) |

^1^ *I*^2^ > 50%

^2^ Publication bias was existed

^3^ Total number of events is less than 300

^4^ The total number of patients is less than 400

^5^ Total number of events is less than 100
