# Supplementary material for: A comparative evaluation of dexmedetomidine and midazolam in pediatric sedation: A meta‐analysis of randomized controlled trials with trial sequential analysis
Source: CNS Neurosci Ther. 2020 Apr 29;26(8):862–75. doi: 10.1111/cns.13377 (PMC7366749; doi:10.1111/cns.13377)
Supplement: Supplementary file 5 — Supplementary Material [file CNS-26-862-s005.docx]

**Figure S1 Flow chart of literature screening and the selection process.**

**Figure S2 Risk of bias assessment of included studies.**

Notes: Green + dot, low risk of bias; yellow ? dot, unclear risk of bias; red - dot, high risk of bias.
